# Supplementary material for: Qualification programmes for immigrant health professionals: A systematic review
Source: PLoS One. 2019 Nov 15;14(11):e0224933. doi: 10.1371/journal.pone.0224933 (PMC6857917; doi:10.1371/journal.pone.0224933)
Supplement: S1 File — (DOCX) [file pone.0224933.s001.docx]

S1 File. Search strings

PUBMED (03.08.2017)

**S1: immigrants/population**

("refugees"[MeSH] OR "refugees"[ALL] OR "refugee"[ALL]) OR ("transients and migrants"[MeSH] OR ("transients"[ALL] AND "migrants"[ALL]) OR "transients and migrants"[ALL] OR "migrants"[ALL] OR ("transient"[ALL] AND "migrant"[ALL])) OR ("emigrants and immigrants"[MeSH] OR ("emigrants"[ALL] AND "immigrants"[ALL]) OR "emigrants and immigrants"[ALL] OR "immigrant"[ALL]) OR ("emigrants and immigrants"[MeSH] OR ("emigrants"[ALL] AND "immigrants"[ALL]) OR "emigrants and immigrants"[ALL] OR "emigrant"[ALL]) OR (immigrated[ALL]) OR (international[ALL]) OR ("internationality"[MeSH] OR "internationality"[ALL] OR "foreign"[ALL]) OR ("transients and migrants"[MeSH] OR ("transients"[ALL] AND "migrants"[ALL]) OR "transients and migrants"[ALL] OR "transient"[ALL]) OR (overseas[ALL] OR oversea[ALL]) OR ("transients and migrants"[MeSH] OR ("transients"[ALL] AND "migrants"[ALL]) OR "transients and migrants"[ALL] OR "nomad"[ALL] or “nomads”[ALL]) OR ("refugees"[MeSH] OR "refugees"[ALL] OR ("asylum"[ALL] AND "seeker"[ALL]) OR "asylum seeker"[ALL] OR ("asylum"[ALL] AND "seekers"[ALL]) OR "asylum seekers"[ALL]) OR ("emigrants and immigrants"[MeSH] OR ("emigrants"[ALL] AND "immigrants"[ALL]) OR "emigrants and immigrants"[ALL] OR "alien"[ALL] OR “aliens”[ALL]) OR (emigrated[ALL]) OR (foreign-born[ALL]) OR ("human migration"[MeSH] OR ("human"[ALL] AND "migration"[ALL]) OR "human migration"[ALL])

AND

**S2: health care professionals/population**

("health personnel"[MeSH] OR ("health"[ALL] AND "personnel"[ALL]) OR "health personnel"[ALL] OR ("health"[ALL] AND "professional"[ALL]) OR "health professional"[ALL] OR “health professionals”[ALL]) OR ("allied health personnel"[MeSH] OR ("allied"[ALL] AND "health"[ALL] AND "personnel"[ALL]) OR "allied health personnel"[ALL] OR ("allied"[ALL] AND "health"[ALL] AND "professional"[ALL]) OR "allied health professional"[ALL] OR “allied health professionals”[ALL]) OR ("health personnel"[MeSH] OR ("health"[ALL] AND "personnel"[ALL]) OR "health personnel"[ALL] OR ("medical"[ALL] AND "personnel"[ALL]) OR "medical personnel"[ALL]) OR ("health personnel"[MeSH] OR ("health"[ALL] AND "personnel"[ALL]) OR "health personnel"[ALL]) OR (("health"[MeSH] OR "health"[ALL]) AND graduate[ALL] OR "health"[ALL] AND graduates[ALL]) OR (medical[ALL] AND graduate[ALL] OR medical[ALL] AND graduates[ALL]) OR ("health personnel"[MeSH] OR ("health"[ALL] AND "personnel"[ALL]) OR "health personnel"[ALL] OR ("health"[ALL] AND "care"[ALL] AND "providers"[ALL]) OR "health care providers"[ALL] OR "health care provider"[ALL]) OR (("counselors"[MeSH] OR "counselors"[ALL] OR "professional"[ALL]) AND ("manpower"[Subheading] OR "manpower"[ALL] OR "personnel"[ALL] OR "research personnel"[MeSH] OR ("research"[ALL] AND "personnel"[ALL]) OR "research personnel"[ALL])) OR (("nursing"[Subheading] OR "nursing"[ALL] OR ("home"[ALL] AND "care"[ALL]) OR "home care"[ALL] OR "home care services"[MeSH] OR ("home"[ALL] AND "care"[ALL] AND "services"[ALL]) OR "home care services"[ALL] OR ("home"[ALL] AND "care"[ALL])) AND ("manpower"[Subheading] OR "manpower"[ALL] OR "personnel"[ALL] OR "research personnel"[MeSH] OR ("research"[ALL] AND "personnel"[ALL]) OR "research personnel"[ALL]))

AND

**S3: intervention**

("Intervention (Amstelveen)"[Journal] OR "intervention"[ALL] OR "Interv Sch Clin"[Journal] OR "intervention"[ALL] OR “interventions”[ALL]) OR (programme[ALL] OR programmes[ALL]) OR (program[ALL] OR programs[ALL]) OR ("education"[Subheading] OR "education"[ALL] OR "training"[ALL] OR "education"[MeSH] OR "training"[ALL] OR “trainings”[ALL]) OR ("training programme"[ALL] OR “training programmes”[ALL] OR "education"[MeSH] OR "education"[ALL] OR ("training"[ALL] AND "program"[ALL]) OR "training program"[ALL] OR “training programs”[ALL]) OR ("education"[Subheading] OR "education"[ALL] OR "teaching"[ALL] OR "teaching"[MeSH]) OR ("vocational education"[MeSH] OR ("vocational"[ALL] AND "education"[ALL]) OR "vocational education"[ALL] OR ("vocational"[ALL] AND "training"[ALL]) OR "vocational training"[ALL]) OR ("education"[Subheading] OR "education"[ALL] OR "educational status"[MeSH] OR ("educational"[ALL] AND "status"[ALL]) OR "educational status"[ALL] OR "education"[ALL] OR "education"[MeSH]) OR (educational[ALL] AND training[ALL] OR educational[ALL] AND trainings[ALL]) OR (educational[ALL] AND program[ALL] OR educational[ALL] AND programs[ALL]) OR (educational[ALL] AND programme[ALL] OR educational[ALL] AND programmes[ALL]) OR ((("acclimatization"[MeSH] OR "acclimatization"[ALL] OR "adaption"[ALL]) AND program[ALL] OR "adaption"[ALL]) AND programs[ALL]) OR ((("acclimatization"[MeSH] OR "acclimatization"[ALL] OR "adaption"[ALL]) AND programme[ALL] OR "adaption"[ALL]) AND programmes[ALL] ) OR (induction[ALL] AND program[ALL] OR induction[ALL] AND programs[ALL]) OR (induction[ALL] AND programme[ALL] OR induction[ALL] AND programmes[ALL]) OR (job-related[ALL] AND ("education"[Subheading] OR "education"[ALL] OR "training"[ALL] OR “trainings”[ALL] OR "education"[MeSH] OR "training"[ALL] OR “trainings”[ALL])) OR (career[ALL] AND program[ALL] OR career[ALL] AND programs[ALL]) OR (career[ALL] AND programme[ALL] OR career[ALL] AND programmes[ALL]) OR (career[ALL] AND ("education"[Subheading] OR "education"[ALL] OR "training"[ALL] OR "education"[MeSH] OR "trainings"[ALL])) OR ("vocational guidance"[MeSH] OR ("vocational"[ALL] AND "guidance"[ALL]) OR "vocational guidance"[ALL]) OR ("education"[Subheading] OR "education"[ALL] OR "curriculum"[ALL] OR "curriculum"[MeSH]) OR (best[ALL] AND practise[ALL]) OR (brief[ALL] AND ("education"[Subheading] OR "education"[ALL] OR "training"[ALL] OR "education"[MeSH] OR "trainings"[ALL])) OR (class[ALL] or classes[ALL]) OR (online[ALL] AND ("education"[Subheading] OR "education"[ALL] OR "training"[ALL] OR "education"[MeSH] OR "trainings"[ALL])) OR ((("counselors"[MeSH] OR "counselors"[ALL] OR "professional"[ALL]) AND skills[ALL] OR "professional"[ALL]) AND skill[ALL]) OR (course[ALL] or courses[ALL]) OR (professionalization[ALL])

AND

**S4: context/health care**

(care[ALL]) OR ("health services"[MeSH] OR ("health"[ALL] AND "services"[ALL]) OR "health services"[ALL] OR "health service"[ALL]) OR (("Appl Catal A Gen"[Journal] OR "general"[ALL]) AND care[ALL]) OR ("delivery of health care"[MeSH] OR ("delivery"[ALL] AND "health"[ALL] AND "care"[ALL]) OR "delivery of health care"[ALL] OR ("health"[ALL] AND "care"[ALL]) OR "health care"[ALL]) OR ("health services"[MeSH] OR ("health"[ALL] AND "services"[ALL]) OR "health services"[ALL] OR ("health"[ALL] AND "care"[ALL] AND "services"[ALL]) OR "health care services"[ALL] OR "health care services"[ALL]) OR ("hospitals"[MeSH] OR "hospitals"[ALL] OR "hospital"[ALL]) OR ("primary health care"[MeSH] OR ("primary"[ALL] AND "health"[ALL] AND "care"[ALL]) OR "primary health care"[ALL]) OR (("delivery of health care"[MeSH] OR ("delivery"[ALL] AND "health"[ALL] AND "care"[ALL]) OR "delivery of health care"[ALL] OR ("health"[ALL] AND "care"[ALL]) OR "health care"[ALL])) OR (tertiary[ALL] AND ("delivery of health care"[MeSH] OR ("delivery"[ALL] AND "health"[ALL] AND "care"[ALL]) OR "delivery of health care"[ALL] OR ("health"[ALL] AND "care"[ALL]) OR "health care"[ALL]))

AND

**S5: outcome/evaluation**

("evaluation studies"[Publication Type] OR "evaluation studies as topic"[MeSH] OR "evaluation"[ALL]) OR ("growth and development"[Subheading] OR ("growth"[ALL] AND "development"[ALL]) OR "growth and development"[ALL] OR "development"[ALL]) OR (outcome[ALL]) OR (effectiveness[ALL]) OR (effect[ALL] OR effects[ALL]) OR (result[ALL] OR results[ALL])

PUBMED (Mesh terms) (03.08.2017)

**S1: immigrants/population**

"Refugees"[Mesh] OR "Transients and Migrants"[Mesh] OR "Emigrants and Immigrants"[Mesh] OR "Internationality"[Mesh] OR "Nurses, International"[Mesh] OR "Human Migration"[Mesh]

AND

**S2: health care professionals/population**

"Health Personnel"[Mesh] OR "Education, Public Health Professional"[Mesh] OR "Allied Health Personnel"[Mesh] OR "Education, Medical, Graduate"[Mesh] OR "Foreign Medical Graduates"[Mesh] OR "Foreign Professional Personnel"[Mesh]

AND

**S3: intervention**

"Early Intervention (Education)"[Mesh] OR "Program Development"[Mesh] OR "Program Evaluation"[Mesh] OR "Education"[Mesh] OR "education" [Subheading] OR "Education, Nursing, Diploma Programs"[Mesh] OR "Education, Nursing, Associate"[Mesh] OR "Teaching"[Mesh] OR "Vocational Education"[Mesh] OR "Program Development"[Mesh] OR "Inservice Training"[Mesh] OR "Vocational Guidance"[Mesh] OR "Curriculum"[Mesh] OR "Education, Professional, Retraining"[Mesh] OR "Education, Distance"[Mesh] OR "Education, Professional"[Mesh]

AND

**S4: context/health care**

"Health Services"[Mesh] OR "Delivery of Health Care"[Mesh] OR "Health Care Sector"[Mesh] OR "Delivery of Health Care, Integrated"[Mesh] OR "Primary Health Care"[Mesh] OR "Home Health Nursing"[Mesh] OR "Hospitals, General"[Mesh] R "General Practice"[Mesh] OR "General Practice, Dental"[Mesh] OR "Health Care Sector"[Mesh] OR "Home Nursing"[Mesh]

AND

**S5: outcome/evaluation**

"Evaluation Studies as Topic"[Mesh] OR "Evaluation Studies" [Publication Type] OR "Program Evaluation"[Mesh] OR "Program Development"[Mesh]

PUBMED (23.09.2019)

**S1: immigrants/population**

("refugees"[MeSH] OR "refugees"[ALL] OR "refugee"[ALL]) OR ("transients and migrants"[MeSH] OR ("transients"[ALL] AND "migrants"[ALL]) OR "transients and migrants"[ALL] OR "migrants"[ALL] OR ("transient"[ALL] AND "migrant"[ALL])) OR ("emigrants and immigrants"[MeSH] OR ("emigrants"[ALL] AND "immigrants"[ALL]) OR "emigrants and immigrants"[ALL] OR "immigrant"[ALL]) OR ("emigrants and immigrants"[MeSH] OR ("emigrants"[ALL] AND "immigrants"[ALL]) OR "emigrants and immigrants"[ALL] OR "emigrant"[ALL]) OR (immigrated[ALL]) OR (international[ALL]) OR ("internationality"[MeSH] OR "internationality"[ALL] OR "foreign"[ALL]) OR ("transients and migrants"[MeSH] OR ("transients"[ALL] AND "migrants"[ALL]) OR "transients and migrants"[ALL] OR "transient"[ALL]) OR (overseas[ALL] OR oversea[ALL]) OR ("transients and migrants"[MeSH] OR ("transients"[ALL] AND "migrants"[ALL]) OR "transients and migrants"[ALL] OR "nomad"[ALL] or “nomads”[ALL]) OR ("refugees"[MeSH] OR "refugees"[ALL] OR ("asylum"[ALL] AND "seeker"[ALL]) OR "asylum seeker"[ALL] OR ("asylum"[ALL] AND "seekers"[ALL]) OR "asylum seekers"[ALL]) OR ("emigrants and immigrants"[MeSH] OR ("emigrants"[ALL] AND "immigrants"[ALL]) OR "emigrants and immigrants"[ALL] OR "alien"[ALL] OR “aliens”[ALL]) OR (emigrated[ALL]) OR (foreign-born[ALL]) OR ("human migration"[MeSH] OR ("human"[ALL] AND "migration"[ALL]) OR "human migration"[ALL])

AND

**S2: health care professionals/population**

("health personnel"[MeSH] OR ("health"[ALL] AND "personnel"[ALL]) OR "health personnel"[ALL] OR ("health"[ALL] AND "professional"[ALL]) OR "health professional"[ALL] OR “health professionals”[ALL]) OR ("allied health personnel"[MeSH] OR ("allied"[ALL] AND "health"[ALL] AND "personnel"[ALL]) OR "allied health personnel"[ALL] OR ("allied"[ALL] AND "health"[ALL] AND "professional"[ALL]) OR "allied health professional"[ALL] OR “allied health professionals”[ALL]) OR ("health personnel"[MeSH] OR ("health"[ALL] AND "personnel"[ALL]) OR "health personnel"[ALL] OR ("medical"[ALL] AND "personnel"[ALL]) OR "medical personnel"[ALL]) OR ("health personnel"[MeSH] OR ("health"[ALL] AND "personnel"[ALL]) OR "health personnel"[ALL]) OR (("health"[MeSH] OR "health"[ALL]) AND graduate[ALL] OR "health"[ALL] AND graduates[ALL]) OR (medical[ALL] AND graduate[ALL] OR medical[ALL] AND graduates[ALL]) OR ("health personnel"[MeSH] OR ("health"[ALL] AND "personnel"[ALL]) OR "health personnel"[ALL] OR ("health"[ALL] AND "care"[ALL] AND "providers"[ALL]) OR "health care providers"[ALL] OR "health care provider"[ALL]) OR (("counselors"[MeSH] OR "counselors"[ALL] OR "professional"[ALL]) AND ("manpower"[Subheading] OR "manpower"[ALL] OR "personnel"[ALL] OR "research personnel"[MeSH] OR ("research"[ALL] AND "personnel"[ALL]) OR "research personnel"[ALL])) OR (("nursing"[Subheading] OR "nursing"[ALL] OR ("home"[ALL] AND "care"[ALL]) OR "home care"[ALL] OR "home care services"[MeSH] OR ("home"[ALL] AND "care"[ALL] AND "services"[ALL]) OR "home care services"[ALL] OR ("home"[ALL] AND "care"[ALL])) AND ("manpower"[Subheading] OR "manpower"[ALL] OR "personnel"[ALL] OR "research personnel"[MeSH] OR ("research"[ALL] AND "personnel"[ALL]) OR "research personnel"[ALL]))

AND

**S3: intervention**

("Intervention (Amstelveen)"[Journal] OR "intervention"[ALL] OR "Interv Sch Clin"[Journal] OR "intervention"[ALL] OR “interventions”[ALL]) OR (programme[ALL] OR programmes[ALL]) OR (program[ALL] OR programs[ALL]) OR ("education"[Subheading] OR "education"[ALL] OR "training"[ALL] OR "education"[MeSH] OR "training"[ALL] OR “trainings”[ALL]) OR ("training programme"[ALL] OR “training programmes”[ALL] OR "education"[MeSH] OR "education"[ALL] OR ("training"[ALL] AND "program"[ALL]) OR "training program"[ALL] OR “training programs”[ALL]) OR ("education"[Subheading] OR "education"[ALL] OR "teaching"[ALL] OR "teaching"[MeSH]) OR ("vocational education"[MeSH] OR ("vocational"[ALL] AND "education"[ALL]) OR "vocational education"[ALL] OR ("vocational"[ALL] AND "training"[ALL]) OR "vocational training"[ALL]) OR ("education"[Subheading] OR "education"[ALL] OR "educational status"[MeSH] OR ("educational"[ALL] AND "status"[ALL]) OR "educational status"[ALL] OR "education"[ALL] OR "education"[MeSH]) OR (educational[ALL] AND training[ALL] OR educational[ALL] AND trainings[ALL]) OR (educational[ALL] AND program[ALL] OR educational[ALL] AND programs[ALL]) OR (educational[ALL] AND programme[ALL] OR educational[ALL] AND programmes[ALL]) OR ((("acclimatization"[MeSH] OR "acclimatization"[ALL] OR "adaption"[ALL]) AND program[ALL] OR "adaption"[ALL]) AND programs[ALL]) OR ((("acclimatization"[MeSH] OR "acclimatization"[ALL] OR "adaption"[ALL]) AND programme[ALL] OR "adaption"[ALL]) AND programmes[ALL] ) OR (induction[ALL] AND program[ALL] OR induction[ALL] AND programs[ALL]) OR (induction[ALL] AND programme[ALL] OR induction[ALL] AND programmes[ALL]) OR (job-related[ALL] AND ("education"[Subheading] OR "education"[ALL] OR "training"[ALL] OR “trainings”[ALL] OR "education"[MeSH] OR "training"[ALL] OR “trainings”[ALL])) OR (career[ALL] AND program[ALL] OR career[ALL] AND programs[ALL]) OR (career[ALL] AND programme[ALL] OR career[ALL] AND programmes[ALL]) OR (career[ALL] AND ("education"[Subheading] OR "education"[ALL] OR "training"[ALL] OR "education"[MeSH] OR "trainings"[ALL])) OR ("vocational guidance"[MeSH] OR ("vocational"[ALL] AND "guidance"[ALL]) OR "vocational guidance"[ALL]) OR ("education"[Subheading] OR "education"[ALL] OR "curriculum"[ALL] OR "curriculum"[MeSH]) OR (best[ALL] AND practise[ALL]) OR (brief[ALL] AND ("education"[Subheading] OR "education"[ALL] OR "training"[ALL] OR "education"[MeSH] OR "trainings"[ALL])) OR (class[ALL] or classes[ALL]) OR (online[ALL] AND ("education"[Subheading] OR "education"[ALL] OR "training"[ALL] OR "education"[MeSH] OR "trainings"[ALL])) OR ((("counselors"[MeSH] OR "counselors"[ALL] OR "professional"[ALL]) AND skills[ALL] OR "professional"[ALL]) AND skill[ALL]) OR (course[ALL] or courses[ALL]) OR (professionalization[ALL])

AND

**S4: context/health care**

(care[ALL]) OR ("health services"[MeSH] OR ("health"[ALL] AND "services"[ALL]) OR "health services"[ALL] OR "health service"[ALL]) OR (("Appl Catal A Gen"[Journal] OR "general"[ALL]) AND care[ALL]) OR ("delivery of health care"[MeSH] OR ("delivery"[ALL] AND "health"[ALL] AND "care"[ALL]) OR "delivery of health care"[ALL] OR ("health"[ALL] AND "care"[ALL]) OR "health care"[ALL]) OR ("health services"[MeSH] OR ("health"[ALL] AND "services"[ALL]) OR "health services"[ALL] OR ("health"[ALL] AND "care"[ALL] AND "services"[ALL]) OR "health care services"[ALL] OR "health care services"[ALL]) OR ("hospitals"[MeSH] OR "hospitals"[ALL] OR "hospital"[ALL]) OR ("primary health care"[MeSH] OR ("primary"[ALL] AND "health"[ALL] AND "care"[ALL]) OR "primary health care"[ALL]) OR (("delivery of health care"[MeSH] OR ("delivery"[ALL] AND "health"[ALL] AND "care"[ALL]) OR "delivery of health care"[ALL] OR ("health"[ALL] AND "care"[ALL]) OR "health care"[ALL])) OR (tertiary[ALL] AND ("delivery of health care"[MeSH] OR ("delivery"[ALL] AND "health"[ALL] AND "care"[ALL]) OR "delivery of health care"[ALL] OR ("health"[ALL] AND "care"[ALL]) OR "health care"[ALL]))

AND

**S5: outcome/evaluation**

("evaluation studies"[Publication Type] OR "evaluation studies as topic"[MeSH] OR "evaluation"[ALL]) OR ("growth and development"[Subheading] OR ("growth"[ALL] AND "development"[ALL]) OR "growth and development"[ALL] OR "development"[ALL]) OR (outcome[ALL]) OR (effectiveness[ALL]) OR (effect[ALL] OR effects[ALL]) OR (result[ALL] OR results[ALL])

🡪 Filters activated: Evaluation Studies

Web of Science (03.08.2017 + 23.09.2019)

**S1 immigrants/population:**

TS=”refugees” OR TS=”refugee” OR TS=”migrant” OR TS=”migrants” OR TS=”immigrant” OR TS=”immigrants” OR TS=”emigrant” OR TS=emigrants” OR TS=”immigrated” OR TS=”international” OR TS=”foreign” OR TS=”transient” OR TS=”transients” OR TS=”overseas” OR TS=”oversea” OR TS=”nomad” OR TS=”nomads” OR TS=”asylum seeker” OR TS=asylum seekers” OR TS=”alien” OR TS=”emigrated” OR TS=”foreign born” OR TS=”human migration”

AND

**S2 health professionals/population:**

TS=”health professional” OR TS=”health professionals” OR TS=”health personnel” OR TS=”medical personnel” OR TS=”health graduate” OR TS=”medical graduate” OR TS=”health care providers” OR TS=”health graduates” OR TS=”medical graduates” OR TS=”health care provider” OR TS=”professional personnel” OR TS=”allied health professional” OR TS=”allied health professionals” OR TS=”home care personnel” OR TS=”caregivers”

AND

**S3 intervention:**

TS=”intervention” OR TS=”interventions” OR TS=”programme” OR TS=”programmes” OR TS=”training” OR TS=”trainings” OR TS=”training programme” OR TS=”training programmes” OR TS=”program” OR TS=”programs” OR TS=”training program” OR TS=”training programs” OR TS=”teaching” OR TS=”vocational training” OR TS=”vocational trainings” OR TS=”education” OR TS=”educational training” OR TS=”educational trainings” OR TS=”educational programme” OR TS=”educational program” OR TS=”educational programmes” OR TS=”educational programs” OR TS=”adaption programme” OR TS=”adaption program” OR TS=”induction programme” OR TS=”induction program” OR TS=”job-related training” OR TS=”adaption programmes” OR TS=”adaption programs” OR TS=”induction programmes” OR TS=”induction programs” OR TS=”job-related trainings” OR TS=”career” OR TS=”career program” OR TS=”career programme” OR TS=” career training” OR TS=”career programs” OR TS=”career programmes” OR TS=” career trainings” OR TS=”vocational guidance” OR TS=”curriculum” OR TS=”best practise” OR TS=”brief training” OR TS=”brief trainings” OR TS=”class” OR TS=”classes” OR TS=”online training” OR TS=”online trainings” OR TS=”professional skills” OR TS=”course” OR TS=”courses” OR TS=”professionalization”

AND

**S4 context/health care:**

TS=”health care” OR TS=”care” OR TS=”health services” OR TS=”health care services” OR TS=”hospital” OR TS=”health service” OR TS=”health care service” OR TS=”hospitals” OR TS=”primary health care” OR TS=” secondary health care” OR TS=”tertiary health care” OR TS=”delivery of health care” OR TS=”health care sector” OR TS=”general practise”

AND

## **S5 outcome:**

## TS=”evaluation” OR TS=”development” OR TS=”outcome” OR TS=”effectiveness” OR TS=”effect” OR TS=”result” OR TS=”effects” OR TS=”results”

CINAHL (03.08.2017 + 23.09.2019)

**S 1 – Immigrants/population**:

refugee OR refugees OR migrant OR migrants OR immigrant OR immigrants OR emigrant OR emigrants OR immigrated OR international OR foreign OR transient OR transients OR overseas OR oversea OR nomad OR nomads OR asylum seekers OR asylum seeker OR alien OR emigrated OR foreign-born OR human migration

AND

**S2 – health professionals/population:**

health professional OR health professionals OR health personnel OR medical personnel OR health graduate OR medical graduate OR health care providers OR health graduates OR medical graduates OR health care provider OR professional personnel OR allied health professional OR allied health professionals OR home care personnel OR caregivers

AND

**S3 - Intervention:**

intervention OR program OR programme OR training OR training program OR training programme OR teaching OR vocational training OR interventions OR programs OR programmes OR trainings OR training programs OR training programmes OR vocational trainings OR education OR educational training OR educational trainings OR educational program OR educational programme OR adaption program OR adaption programme OR induction program OR induction programme OR job-related training OR educational programs OR educational programmes OR adaption programs OR adaption programmes OR induction programs OR induction programmes OR job-related trainings OR career OR career-program OR career-programme OR career-training OR career-programs OR career-programmes OR career-trainings OR vocational guidance OR curriculum OR best practise OR brief training OR brief trainings OR class OR classes OR online training OR online trainings OR professional skills OR course OR courses OR Professionalisation

AND

**S4 – health care /context**:

care OR health services OR health service OR general care OR health care OR health care services OR health care service OR hospital OR hospitals OR primary health care OR secondary health care OR tertiary health care OR delivery of health care OR health care sector OR general practise

AND

**S5 outcome**/evaluation:

evaluation OR development OR outcome OR effectiveness OR effect OR result OR effects OR results

PsychInfo (04.08.2017 + 23.09.2019)

**S1: Population/Immigrants**

1. refugee.mp. or exp REFUGEES/

2. exp Human Migration/ or exp Immigration/ or migrant.mp.

2. immigrant.mp. or exp Immigration/

4. exp Human Migration/ or exp Immigration/ or emigrant.mp.

5. exp Immigration/ or immigrated.mp.

6. exp INTERNATIONAL STUDENTS/ or international.mp.

7. exp FOREIGN WORKERS/ or exp FOREIGN LANGUAGE LEARNING/ or exp FOREIGN LANGUAGE EDUCATION/ or foreign.mp.

8. transient.mp.

9. exp Expatriates/ or exp Human Migration/ or oversea.mp. or exp International Students/

10. nomad.mp.

11. exp Asylum Seeking/ or exp Refugees/ or asylum seeker.mp.

12. emigrated.mp.

13. foreign born.mp.

14. 1 or 2 or 3 or 4 or 5 or 6 or 7 or 8 or 9 or 10 or 11 or 12 or 13

refugee.mp. or exp REFUGEES/ or exp Human Migration/ or exp Immigration/ or migrant.mp. or migrants.mp. or immigrants.mp. or exp Immigration/ or immigrant.mp. or exp Immigration/ or exp Human Migration/ or exp Immigration/ or emigrant.mp. or emigrants.mp. or exp Immigration/ or immigrated.mp. or exp INTERNATIONAL STUDENTS/ or international.mp. or exp FOREIGN WORKERS/ or foreign worker.mp. or exp FOREIGN LANGUAGE LEARNING/ or exp FOREIGN LANGUAGE EDUCATION/ or foreign.mp. or transient.mp. or exp Expatriates/ or exp Human Migration/ or oversea.mp. or exp International Students/ or nomad.mp. or exp Asylum Seeking/ or exp Refugees/ or asylum seeker.mp. or emigrated.mp. or foreign born.mp.

**S2: Population/Health professionals**

1. exp Health Personnel/ or health professional.mp. Exp health personnel
2. exp Education Students/ or exp Graduate Students/ or exp Medical Students/ Exp medical graduate
3. health care provider.mp. Exp professional personnel
4. medical personnel.mp. or exp Medical Personnel/
5. professional personnel.mp. or exp Professional Personnel/
6. caregivers.mp. or exp CAREGIVERS/
7. exp Allied Health Personnel/ or allied health professional.mp.
8. 1 or 2 or 3 or 4 or 5 or 6 or 7

health personnel.mp. or exp Health Personnel/ OR exp Health Personnel/ or exp Mental Health Personnel/ or health professional.mp. or health professionals.mp. OR exp International Students/ or international student/ or exp Expatriates/ or Expatriate/ or medical graduate.mp. or medical graduates.mp. OR exp Health Care Services/ OR exp Health Care Service/ or exp Health Care Delivery/ or health care provider.mp. OR medical personnel.mp. or exp Medical Personnel/ OR professional personnel.mp. or exp Professional Personnel/ OR caregivers.mp. or exp CAREGIVERS/ OR caregiver.mp. OR exp Allied Health Personnel/ or allied health professional.mp. or allied health professionals.mp.

**S3: Intervention**

1. program.mp. or exp EDUCATIONAL PROGRAM EVALUATION/ or exp PROGRAM DEVELOPMENT/ or exp PROGRAM EVALUATION/
2. exp Educational Programs/ or programme.mp.
3. exp SOCIAL SKILLS TRAINING/ or training.mp. or exp WORK ADJUSTMENT TRAINING/ or exp TRAINING/ or exp PERSONNEL TRAINING/
4. training program.mp. or exp Intervention/
5. training programme.mp.
6. teaching.mp. or exp TEACHING/
7. exp Educational Programs/ or exp Vocational Education/ or exp Occupational Guidance/ or vocational training.mp.
8. exp VOCATIONAL EDUCATION/ or education.mp. or exp EDUCATION/
9. educational training.mp.
10. educational program.mp. or exp Educational Programs/
11. educational programme.mp.
12. adaption program.mp.
13. exp Curriculum Based Assessment/ or adaption programme.mp.
14. induction program.mp.
15. induction programme.mp.
16. exp On the Job Training/ or job related training.mp. Exp career
17. career program.mp.
18. career programme.mp.
19. career training.mp.
20. vocational guidance.mp. or exp Occupational Guidance/
21. exp CURRICULUM BASED ASSESSMENT/ or curriculum.mp. or exp CURRICULUM/ or exp CURRICULUM DEVELOPMENT/
22. best practise.mp.
23. brief training.mp.
24. class.mp.
25. exp Computer Assisted Instruction/ or online training.mp.
26. professional skills.mp.
27. course.mp.
28. exp Professional Development/ or Professionalization.mp.
29. 1 or 2 or 3 or 4 or 5 or 6 or 7 or 8 or 9 or 10 or 11 or 12 or 13 or 14 or 15 or 16 or 17 or 18 or 19 or 20 or 21 or 22 or 23 or 24 or 25 or 26 or 27 or 28

program.mp. or programs.mp. OR exp EDUCATIONAL PROGRAM EVALUATION/ or exp PROGRAM DEVELOPMENT/ or exp PROGRAM EVALUATION/ OR exp Educational Programs/ or programme.mp. or programmes.mp. OR exp SOCIAL SKILLS TRAINING/ or training.mp. or exp WORK ADJUSTMENT TRAINING/ or exp TRAINING/ or exp PERSONNEL TRAINING/ OR training program.mp. or training programmes.mp. or training programs.mp. or exp Intervention/ or interventions.mp. OR training programme.mp. OR teaching.mp. or exp TEACHING/ OR exp Educational Programs/ or exp Vocational Education/ or exp Occupational Guidance/ or vocational training.mp. or vocational training.mp. OR exp VOCATIONAL EDUCATION/ or education.mp. or exp EDUCATION/ OR educational training.mp. OR educational program.mp. or educational programs.mp. or exp Educational Programs/ OR educational programme.mp. or educational programmes.mp. OR adaption program.mp. or adaption programs.mp. OR exp Curriculum Based Assessment/ or adaption programme.mp. or adaption programmes.mp. OR induction program.mp. or induction programs.mp. OR induction programme.mp. or induction programmes.mp. OR exp On the Job Training/ or job related training.mp. or job related trainings.mp. OR career.mp. OR career program.mp. or career programs.mp. OR career programme.mp. or career programmes.mp. OR career training.mp. or career trainings.mp. OR vocational guidance.mp. or exp Occupational Guidance/ OR exp CURRICULUM BASED ASSESSMENT/ or curriculum.mp. or exp CURRICULUM/ or exp CURRICULUM DEVELOPMENT/ OR best practise.mp. OR brief training.mp. or brief trainings.mp. OR class.mp. or classes.mp. OR exp Computer Assisted Instruction/ or online training.mp. or online trainings.mp. OR professional skills.mp. or professional skill.mp. OR course.mp. or courses.mp. OR exp Professional Development/ or Professionalization.mp.

**S4: Context/Health Care**

1. exp PALLIATIVE CARE/ or exp PRIMARY HEALTH CARE/ or care.mp. or exp HEALTH CARE DELIVERY/ or exp INTENSIVE CARE/ or exp HEALTH CARE SERVICES/ or exp LONG TERM CARE/ or exp CHILD CARE/ or exp ADULT DAY CARE/ or exp HOME CARE/
2. exp Health Care Services/ or exp Home Care/ or exp Mental Health Services/ or exp Community Mental Health Services/ or health services.mp. or exp Health Care Delivery/ or exp Hospitals/
3. general care.mp.
4. health care.mp.
5. health care services.mp. or exp Health Care Services/
6. exp PSYCHIATRIC HOSPITALS/ or exp HOSPITALS/ or hospital.mp.
7. primary health care.mp. or exp Primary Health Care/
8. secondary health care.mp.
9. tertiary health care.mp.
10. exp Health Care Delivery/ or delivery of health care.mp.
11. health care sector.mp.
12. exp Clinical Practice/ or exp General Practitioners/ or general practise.mp.
13. 1 or 2 or 3 or 4 or 5 or 6 or 7 or 8 or 9 or 10 or 11 or 12

exp PALLIATIVE CARE/ or exp PRIMARY HEALTH CARE/ or care.mp. or exp HEALTH CARE DELIVERY/ or exp INTENSIVE CARE/ or exp HEALTH CARE SERVICES/ or exp LONG TERM CARE/ or exp CHILD CARE/ or exp ADULT DAY CARE/ or exp HOME CARE/ OR exp Health Care Service/ or exp Home Care/ or exp Mental Health Services/ or exp Community Mental Health Services/ or health services.mp. or health service.mp. or exp Health Care Delivery/ or exp Hospitals/ OR general care.mp. OR health care.mp. OR health care services.mp. or health care service.mp. or exp Health Care Services/ OR exp PSYCHIATRIC HOSPITALS/ or exp HOSPITALS/ or hospital.mp. OR primary health care.mp. or exp Primary Health Care/ OR secondary health care.mp. OR tertiary health care.mp. OR exp Health Care Delivery/ or delivery of health care.mp. OR health care sector.mp. OR exp Clinical Practice/ or exp General Practitioners/ or general practise.mp.

**S5: Outcome/Evaluation**

1. exp COURSE EVALUATION/ or exp EVALUATION/ or exp PROGRAM EVALUATION/ or exp EDUCATIONAL PROGRAM EVALUATION/ or evaluation.mp. or exp VOCATIONAL EVALUATION/
2. exp PROFESSIONAL DEVELOPMENT/ or exp PROGRAM DEVELOPMENT/ or exp CURRICULUM DEVELOPMENT/ or exp DEVELOPMENT/
3. outcome.mp.
4. effectiveness.mp.
5. effect.mp.
6. result.mp.
7. 1 or 2 or 3 or 4 or 5 or 6

exp COURSE EVALUATION/ or exp EVALUATION/ or exp PROGRAM EVALUATION/ or exp EDUCATIONAL PROGRAM EVALUATION/ or evaluation.mp. or exp VOCATIONAL EVALUATION/ OR exp PROFESSIONAL DEVELOPMENT/ or exp PROGRAM DEVELOPMENT/ or exp CURRICULUM DEVELOPMENT/ or exp DEVELOPMENT/ OR outcome.mp. OR effectiveness.mp. OR effect.mp. OR effects.mp. OR result.mp. OR results.mp.

EBSCO (03.08.2017)

S 1 immigrants/population:

refugee OR refugees OR migrant OR migrants OR immigrant OR immigrants OR emigrant OR emigrants OR immigrated OR international OR foreign OR transient OR transients OR overseas OR oversea OR nomad OR nomads OR asylum seekers OR asylum seeker OR alien OR emigrated OR foreign-born OR human migration

AND

S2 health professionals/population:

health professional OR health professionals OR health personnel OR medical personnel OR health graduate OR medical graduate OR health care providers OR health graduates OR medical graduates OR health care provider OR professional personnel OR allied health professional OR allied health professionals OR home care personnel OR caregivers

AND

S3 intervention:

intervention OR program OR programme OR training OR training program OR training programme OR teaching OR vocational training OR interventions OR programs OR programmes OR trainings OR training programs OR training programmes OR vocational trainings OR education OR educational training OR educational trainings OR educational program OR educational programme OR adaption program OR adaption programme OR induction program OR induction programme OR job-related training OR educational programs OR educational programmes OR adaption programs OR adaption programmes OR induction programs OR induction programmes OR job-related trainings OR career OR career-program OR career-programme OR career-training OR career-programs OR career-programmes OR career-trainings OR vocational guidance OR curriculum OR best practise OR brief training OR brief trainings OR class OR classes OR online training OR online trainings OR professional skills OR course OR courses OR Professionalisation

AND

S4 context/health care:

care OR health services OR health service OR general care OR health care OR health care services OR health care service OR hospital OR hospitals OR primary health care OR secondary health care OR tertiary health care OR delivery of health care OR health care sector OR general practise

AND

S5 outcome:

evaluation OR development OR outcome OR effectiveness OR effect OR result OR effects OR results

EBSCO (23.09.2019)

S 1 immigrants/population:

refugee OR refugees OR migrant OR migrants OR immigrant OR immigrants OR emigrant OR emigrants OR immigrated OR international OR foreign OR transient OR transients OR overseas OR oversea OR nomad OR nomads OR asylum seekers OR asylum seeker OR alien OR emigrated OR foreign-born OR human migration

AND

S2 health professionals/population:

health professional OR health professionals OR health personnel OR medical personnel OR health graduate OR medical graduate OR health care providers OR health graduates OR medical graduates OR health care provider OR professional personnel OR allied health professional OR allied health professionals OR home care personnel OR caregivers

AND

S3 intervention:

intervention OR program OR programme OR training OR training program OR training programme OR teaching OR vocational training OR interventions OR programs OR programmes OR trainings OR training programs OR training programmes OR vocational trainings OR education OR educational training OR educational trainings OR educational program OR educational programme OR adaption program OR adaption programme OR induction program OR induction programme OR job-related training OR educational programs OR educational programmes OR adaption programs OR adaption programmes OR induction programs OR induction programmes OR job-related trainings OR career OR career-program OR career-programme OR career-training OR career-programs OR career-programmes OR career-trainings OR vocational guidance OR curriculum OR best practise OR brief training OR brief trainings OR class OR classes OR online training OR online trainings OR professional skills OR course OR courses OR Professionalisation

AND

S4 context/health care:

care OR health services OR health service OR general care OR health care OR health care services OR health care service OR hospital OR hospitals OR primary health care OR secondary health care OR tertiary health care OR delivery of health care OR health care sector OR general practise

AND

S5 outcome: evaluation OR development OR outcome OR effectiveness OR effect OR result OR effects OR results

🡪 Narrow by: medical personnel, study & teaching, medical education

ProQuest Social Sciences (03.08.2017)

S 1: refugee OR refugees OR migrant OR migrants OR immigrant OR immigrants OR emigrant OR emigrants OR immigrated OR international OR foreign OR transient OR transients OR overseas OR oversea OR nomad OR nomads OR asylum seekers OR asylum seeker OR alien OR emigrated OR foreign-born OR human migration

AND

S2: health professional OR health professionals OR health personnel OR medical personnel OR health graduate OR medical graduate OR health care providers OR health graduates OR medical graduates OR health care provider OR professional personnel OR allied health professional OR allied health professionals OR home care personnel OR caregivers

AND

S3: intervention OR program OR programme OR training OR training program OR training programme OR teaching OR vocational training OR interventions OR programs OR programmes OR trainings OR training programs OR training programmes OR vocational trainings OR education OR educational training OR educational trainings OR educational program OR educational programme OR adaption program OR adaption programme OR induction program OR induction programme OR job-related training OR educational programs OR educational programmes OR adaption programs OR adaption programmes OR induction programs OR induction programmes OR job-related trainings OR career OR career-program OR career-programme OR career-training OR career-programs OR career-programmes OR career-trainings OR vocational guidance OR curriculum OR best practise OR brief training OR brief trainings OR class OR classes OR online training OR online trainings OR professional skills OR course OR courses OR Professionalisation

AND

S4: care OR health services OR health service OR general care OR health care OR health care services OR health care service OR hospital OR hospitals OR primary health care OR secondary health care OR tertiary health care OR delivery of health care OR health care sector OR general practise

AND

S5: evaluation OR development OR outcome OR effectiveness OR effect OR result OR effects OR results

ProQuest Social Sciences (23.09.2019)

S 1: refugee OR refugees OR migrant OR migrants OR immigrant OR immigrants OR emigrant OR emigrants OR immigrated OR international OR foreign OR transient OR transients OR overseas OR oversea OR nomad OR nomads OR asylum seekers OR asylum seeker OR alien OR emigrated OR foreign-born OR human migration

AND

S2: health professional OR health professionals OR health personnel OR medical personnel OR health graduate OR medical graduate OR health care providers OR health graduates OR medical graduates OR health care provider OR professional personnel OR allied health professional OR allied health professionals OR home care personnel OR caregivers

AND

S3: intervention OR program OR programme OR training OR training program OR training programme OR teaching OR vocational training OR interventions OR programs OR programmes OR trainings OR training programs OR training programmes OR vocational trainings OR education OR educational training OR educational trainings OR educational program OR educational programme OR adaption program OR adaption programme OR induction program OR induction programme OR job-related training OR educational programs OR educational programmes OR adaption programs OR adaption programmes OR induction programs OR induction programmes OR job-related trainings OR career OR career-program OR career-programme OR career-training OR career-programs OR career-programmes OR career-trainings OR vocational guidance OR curriculum OR best practise OR brief training OR brief trainings OR class OR classes OR online training OR online trainings OR professional skills OR course OR courses OR Professionalisation

AND

S4: care OR health services OR health service OR general care OR health care OR health care services OR health care service OR hospital OR hospitals OR primary health care OR secondary health care OR tertiary health care OR delivery of health care OR health care sector OR general practise

AND

S5: evaluation OR development OR outcome OR effectiveness OR effect OR result OR effects OR results

🡪 Narrow by main subjects, German and English
