# Supplementary material for: Qualification programmes for immigrant health professionals: A systematic review
Source: PLoS One. 2019 Nov 15;14(11):e0224933. doi: 10.1371/journal.pone.0224933 (PMC6857917; doi:10.1371/journal.pone.0224933)
Supplement: S1 Table — (DOCX) [file pone.0224933.s002.docx]

S1 Table. Screening criteria

| First stage of screening | |
| --- | --- |
| Population | immigrant health professionals, refugee health professionals, international, foreign and overseas trained health professionals |
| Intervention | programmes that aimed to prepare the population for working in health professions |
| Second stage of screening | |
| Population | - immigrant health professionals, refugee health professionals, international, foreign and overseas trained health professionals - every health care profession according to the international labour organisation [34] |
| Intervention | - programmes preparing the population for working in health professions - occupational specific educational programmes - programmes focusing on the recognition and licensing of the population - health profession specific language courses - intervention and sample must exceed two days and two participants   Exclusion criteria:   - programmes for groups that are already working in their original occupations |
| Context | - labour market integration into health professions and health context - primary, secondary or tertiary care - contact to patients or with machines in health care |
| Outcome | - qualitative or quantitative evaluations - transparency in terms of evaluation methods |
| Study Design | - studies with primary and secondary data - studies carried out in a qualitative or quantitative manner - studies in German and English   Exclusion criteria:   - Commentaries, newspaper articles, and policy papers |
