# Supplementary material for: Qualification programmes for immigrant health professionals: A systematic review
Source: PLoS One. 2019 Nov 15;14(11):e0224933. doi: 10.1371/journal.pone.0224933 (PMC6857917; doi:10.1371/journal.pone.0224933)
Supplement: S2 Table — (DOCX) [file pone.0224933.s003.docx]

S2 Table. Study characteristics

| Reference | Host Country | Target Population | Study Design | Summary of Intervention | Sample | | Description of Evaluation methods | Summary of Outcomes |
| --- | --- | --- | --- | --- | --- | --- | --- | --- |
|  |  |  |  |  | total N | Age, Gender, Country of origin |  |  |
| Andrew, 2010  [18] | Canada/ Vancouver | International medical graduates (IMGs) | Non-randomised controlled trial | This programme was a family practise residency programme with a specific training site and teaching for IMGS in order to address more cultural, ethical, communication-related issues. | IMGs N = 371  Canadian: N = 313 | Age: M = 40 | In-training evaluation report (ITER) and results of Canadian Certification in Family Medicine (CCFP) examination pass rates between IMGs and control group | There were no significant differences in the In-training evaluation report between Canadian and IMG students. In passing the CCFP examination Canadians still were more successful (95%) than IMGs (58%). |
|  |  |  |  |  |  | Gender: no information |  |  |
|  |  |  |  |  |  | Country of origin: no information |  |  |
| Atack et al., 2012  [20] | Canada/ Ontario | Internationally educated nurses | Qualitative | This programme combined three elements: teaching, online based exercises and practise including English skills and an introduction to professional practise in Canada. | N = 62 (total)   1. Focus group sessions (N = 29)   2.  Focus group (N = 19)  3. telephone interviews (N = 9) | Age: no information | Focus groups and interviews at several points about individual feedback and programme strength and gaps | Programme enhanced participant’s confidence; practise was seen as most valuable in adapting to host country's health care. |
|  |  |  |  |  |  | Gender:  53 female, 9 male |  |  |
|  |  |  |  |  |  | Country of origin: no information |  |  |
| Daniel et al., 2016  [37] | Canada | Internationally educated health professionals (IEHPs) | Incidence Study | This programme introduced a Clinical Practise Facilitator (CPF) during an internship (including weekly classes) of IEHPs. The CPF had multiple roles: giving feedback to IEHPs, represent their interests, guide and encourage them. | N = 35 IEPs N = 37 CIs | Age:  no information | Self-developed questionnaire at the end of programme about the role of the CPF and benefits and challenges of the role | The versatile role of CPF was seen in several ways beneficial as for example to provide feedback, to answer questions and to support participants. Participants perceived cultural differences between the CPF and themselves as challenging. |
|  |  |  |  |  |  | Gender:  no information |  |  |
|  |  |  |  |  |  | Country of origin: mostly Philippines (28%) and India (21%) |  |  |
| Fernández-Peña, 2012  [38] | USA/ California | Immigrant health professionals | Incidence Study | This programme focused on case management, building up networks for migrant health professionals as well as introducing them to US health care practise and language through courses. | N = 10.476 | Age: 60% were between 30 and 49 | Demographic descriptive data (job post, exam taking rates, obtaining license, etc.) | WBI had a wide scope and reached a lot of immigrant health professionals.  Approx. half of them succeeded in some way (validating their credentials, passed their exam, gained employment or higher positions). |
|  |  |  |  |  |  | Gender: 72% female, 28% male |  |  |
|  |  |  |  |  |  | Country of origin: Mexico, Philippines, El Salvador, China, Peru, Colombia, Russia, India, Ukraine, Nicaragua, Iran, Haiti, Brazil, Guatemala |  |  |
| Hawken, 2005 [21] | New Zealand/ Auckland, Wellington | Overseas-trained doctors (OTDs) | Non-randomised controlled trial | This programme combined teaching (consultation, communication, cultural issued and ethics) and supervised clinical practise. | N = 96 | Age: no information | Pre-post and post course self-developed questionnaire for alumni’s about the usefulness of the course, the participants' perception of their skills before and after the programme and suggestions for improvement | There was a significant increase (p<0.001) in participants comfort with their abilities to communicate effectively with patients in particular Maori patients (p<0.001). |
|  |  |  |  |  |  | Gender: 22 female, 74 male |  |  |
|  |  |  |  |  |  | Country of origin: Bangladesh, India, Sri Lanka, China, Egypt, Iraq, Iran, Singapore, Russia, Philippines, Serbia, Albania, Croatia |  |  |
| Lujan & Little 2010 [25] | major city of the United-States | Migrated nurses | Mixed Methods | This programme was based on teaching with a focus on preparing for the state-approved examination. | N = 20 | Age: M = 28 | Formative and summative evaluation through verbal short answers and written examination results | Half of the participants passed the NCLEX-RN test with a pass rate of 50% which is higher than the earlier reported pass rate of 22%. |
|  |  |  |  |  |  | Gender: 19 female, 1 male |  |  |
|  |  |  |  |  |  | Country of origin: Mexico |  |  |
| Majum-dar et al. 1999 [39] | Canada/ Ontario, Toronto | Foreign medical graduates (FMGs) | Non-randomised controlled trial | This programme leaned on teaching through group sessions, simulated interviews and observation of videos focusing on communication and cultural aspects. | N = 24 (experimental group)  N = 24 (control group) | Age: no information | Cross-cultural Adaptability Inventory (CCAI) was used to assess ones effectiveness in cross-cultural situations compared to a control group pre-test/post-test | Significant differences were found in two dimensions: emotional resilience (p<0.001) and perceptual acuity (p<0.03). |
|  |  |  |  |  |  | Gender: 48% female, 52% male |  |  |
|  |  |  |  |  |  | Country of origin: mostly China, Vietnam, Egypt |  |  |
| McGrat & Hender-son, 2009 [40] | Australia/ Queens-land | International medical graduates (IMGs) | Qualitative | This programme facilitated observerships and additional education with medical/ professional knowledge. | N = 9 | Age: range from 30-46 | Post course telephone-interviews about the participants' experiences with the programme, strengths and weaknesses of the programme | Programme was helpful and supportive for participants’ entrance into workforce. Participants reported higher self-confidence, familiarity with the Australian health care and improvement of language and professional skills. |
|  |  |  |  |  |  | Gender: 4 female, 5 male |  |  |
|  |  |  |  |  |  | Country of origin: mostly China (n  =6), Yugoslavia (n=1), Philippines (n=1) and Sri Lanka (n=1) |  |  |
| Ong & Paice, 2006  [15] | United Kingdom | Refugee doctors | Mixed Methods | This programme facilitated ‘Senior House Officer’ posts and introduced National Health Service (NHS) and other relevant issues through induction days. | N = 25 | Age: M = 41 years | Post course questionnaires and group discussions about participants view of the programme were evaluated along with job post rates | Participants reported improved confidence and knowledge. They were able to build networks. 15 of the 25 participants achieved substantive jobs within 12 months. |
|  |  |  |  |  |  | Gender: 9 female, 16 male |  |  |
|  |  |  |  |  |  | Country of origin: mostly Iraq and Iran |  |  |
| Parrone et al., 2008  [26] | USA/  Midwest | Foreign nurses | Non-randomised controlled trial | This programme focused on preparing for the NCLEX-RN Examination and developing practical skills in a laboratory. Counselling and tutoring was provided when needed. | N = 67 | Age: range from 23-58 | Descriptive data about the correlation between attending the course, scoring rates of the HESI examinations and passing rates in the NCLEX-RN examination | There was a significant correlation (p<0.05) between HESI scores and NCLEX-RN pass rates. |
|  |  |  |  |  |  | Gender: 52 female, 15 male |  |  |
|  |  |  |  |  |  | Country of origin: mostly Philippines |  |  |
| Peters & Braeseke, 2016 [19] | Germany | Immigrant nurses | Mixed Methods | This programme consisted of theoretical (language, work, care) and practical training prior to a language and intercultural training at arrival. | N= 138 (interviews)  N = 100 (questionnaires) | Age: no information | Formative and summative evaluation through (group) Interviews and self-developed questionnaires about the participants' and facilities' experiences | The majority (92%) of the nurses completed the training and now work as nurses. Nurses were satisfied with the programme. Over 90% of the nurses approved the usefulness of intercultural training. |
|  |  |  |  |  |  | No information |  |  |
|  |  |  |  |  |  | Country of origin: Vietnam |  |  |
| Sullivan et al., 2002, [41] | Australia/ New South Wales | Overseas-trained doctors (OTDs) | Non-randomised controlled trial | This programme combined teaching and supervised clinical attachment. | N = 66 | Age: female M = 37, male M = 36 | Self-developed pre and post-test questionnaires, satisfaction sheets for daily sessions and a focus group at the end of the programme | Participants gained more confidence in their abilities to cope (p<0.004) and in relating with patients and peers (p<0,000), their communication (p<0.000) and their judgemental (p<0.046) skills. Participants had a greater understanding of the system and were less concerned about getting back to work. |
|  |  |  |  |  |  | Gender: 58% female, 42% male |  |  |
|  |  |  |  |  |  | Country of origin: no information |  |  |
| Wright et al., 2011 [42] | Australia/ Gippsland | International medical graduates (IMGs) | Mixed Methods | This programme offered simulated consultations along with meetings and web-based educational tools and a short period of observed practise. | N = 17 | Age: M = 35 | Self-developed questionnaires on meeting the learning objectives, pre post self- and external assessment through multisource feedback (MSF) and telephone interviews after the programme | Significant improvement was identified in three areas: technical skills, willingness and  effectiveness when teaching colleagues and communication with carers and family. |
|  |  |  |  |  |  | Gender: 7 female, 10 male |  |  |
|  |  |  |  |  |  | Country of origin: Sri Lanka, the Philippines, Colombia, India, Bulgaria, Bangladesh, Iran, Afghanistan, Vietnam, China, Egypt and Bosnia |  |  |
| Baker & Robson, 2012  [43] | United Kingdom/ Scotland/ Dumfries and Galloway | International medical graduates (IMGs) | Mixed Methods | This programme focused on teaching language and consultation skills. | N = 14 | Age: no information | Pre-post language skills assessment and post course focus groups | There was a significant improvement in defining clinical problems (p<0. 02) and explaining problems (p<0.004) to patients. 44% of the supervisors saw an improvement in language and consultation skills. |
|  |  |  |  |  |  | Gender: no information |  |  |
|  |  |  |  |  |  | Country of origin: India (n = 9), Pakistan (n = 2), Sri Lanka (n = 1), Libya (n = 1), Sudan (n =1) |  |  |
| Bruce et al., 1974 [44] | United States/ Illinois | Foreign medical graduates (FMGs) | Non-randomised controlled trial | This programme was a language course designed for the needs of FMGs in speaking publicly. | N = 9 | Age: no information | Pre-post audio and video language assessment | The scores on audio and video performance before and after the programme showed significant improvement (p<0.005). |
|  |  |  |  |  |  | Gender: 5 female, 4 male |  |  |
|  |  |  |  |  |  | Country of origin: Korea (n = 5), Philippines (n = 1), Taiwan (n = 1), Egypt (n = 1), Iran (n = 1) |  |  |
| Cheung 2011  [45] | United Kingdom | Overseas-trained doctors (OTDs) | Mixed Methods | This programme combined teaching, peer support through other staff members and professional advice on career if needed. | N = 12 | Age: no information | Post course (self-developed) questionnaire, focus group and telephone interviews | Participants rated the course as relevant (M = 4.7 on a 5-point Likert scale), adequate (M = 4.2). Participants highlighted the peer support especially when the peers had the same cultural background as the participants. |
|  |  |  |  |  |  | Gender: no information |  |  |
|  |  |  |  |  |  | Country of origin: no information |  |  |
| Elis et al., 2005  [22] | Israel | Foreign graduate residents | Non-randomised controlled trial | This programme focused on teaching medical subspecialties and preparation for examinations. | Study group: N = 130 internal medicine residents; Control group: N = 405 residents | Age: range 28-53 | Self-developed feedback questionnaire post course, results in the Israeli examination compared to a control group | A high overall satisfaction score was given by the participants in response to the course (M = 4.28 on a 5-point Likert scale). Participants of the course had a significant higher chance of passing than the ones in the control group (41,7% vs. 30,4%; p<0.001). |
|  |  |  |  |  |  | Gender Study group: 74 female, 56 males |  |  |
|  |  |  |  |  |  | Country of origin: mostly Soviet Union |  |  |
| Gerrish & Griffith, 2004 [46] | United Kingdom | Overseas registered nurses | Qualitative | This programme combined three elements: an induction period, a supervised clinical practise and a mentorship by other nurses. Additional support was provided if needed. | N= 17 | Age: no information | Individual and focus group interviews at several times | Participants identified areas of success they connected to the programme which were most important to them (such as gaining professional registration, fitness for practise, getting employed and professional development in a valued organisational culture). |
|  |  |  |  |  |  | Gender: 17 female |  |  |
|  |  |  |  |  |  | Country of origin: China, Philippines, India, sub-Saharan Africa |  |  |
| Goldszmidt et al., 2007 [47] | Canada | International medical graduates (IMGs) and Internationally sponsored residents (ISRs) | Non-randomised controlled trial | This programme focused on English for medical purposes thus learning through clinical standardised patient scenarios. | ISRs N = 5,  IMG N = 1 | Age: no information | Post programme feedback and pre-post self-evaluation of their skills | There was a significant increase in their communication skills  (p = 0. 03). |
|  |  |  |  |  |  | Gender: no information |  |  |
|  |  |  |  |  |  | Country of origin: no information |  |  |
| Greig et al., 2013 [23] | Canada | Internationally educated physiotherapists (IEPs) | Mixed Methods | This programme combined teaching (medical subjects and preparation for examinations) and a mentorship. | IEPs N = 124 | Age: no information | National exam results between control and intervention group | More than half of the participants (69/124) were integrated into workforce after the programme. Participation led to a 28% greater possibility of passing the written examination. |
|  |  |  |  |  |  | Gender: no information |  |  |
|  |  |  |  |  |  | Country of origin: UK (31%), India (21%), Australia (12%), Philippines (7%), US (5%), Brazil (5%), Iran (4%), Israel (3%), Netherlands (3%) |  |  |
| Harris & Delany, 2013 [14] | Australia/ Victoria | International medical graduates (IMGs) | Qualitative | This programme facilitated discussion and reflection sessions between IMGS and hospital staff. | No information | Age: no information | Feedback through evaluation cards after each session | Participants reported better adjustments to their new workplace and encouragement to critically reflect differences between their previous and current workplaces. |
|  |  |  |  |  |  | Gender: no information |  |  |
|  |  |  |  |  |  | Country of origin: no information |  |  |
| Horner, 2004 [48] | United Kingdom | Internationally recruited nurses | Non-randomised controlled trial | This programme facilitated a supervised practise programme. | IRNs N = 460 Mentors N = 100 | Age: no information | Self-developed post course questionnaire | Most of the participants that responded (response rate 23%) evaluated the programme as very beneficial and highlighted that having a mentor or some kind of support was important. Study days increased their confidence and knowledge. |
|  |  |  |  |  |  | Gender: no information |  |  |
|  |  |  |  |  |  | Country of origin: mostly from Philippines and Singapore |  |  |
| Lax et al., 2009 [49] | Canada/ Toronto | International medical graduates (IMGs) | Incidence Study | This programme consisted of a web-based e-learning programme focusing on communication and cultural issues through simulated doctor/patient scenarios, knowledge checks, reflective exercises and cases about medical topics. | S1: N = 20 S2: N = 42  S3: N = 33 | Age: no information | Usability test through a self-developed questionnaire and monitoring of participants' use of the web-based programme | Participants showed high levels of participation in the programme. Repeated participation and revision indicated knowledge building. |
|  |  |  |  |  |  | Gender: no information |  |  |
|  |  |  |  |  |  | Country of origin: no information |  |  |
| Ong et al., 2002 [50] | United Kingdom/ London | Overseas-trained doctors (OTDs) | Non-randomised controlled trial | This programme offered teaching courses on several topics such as communication, professional practise and health care system, multicultural issues and job searching skills. | N = 136 | Age: no information | Self-developed questionnaire after every daily session about the usefulness of the session | Topics were generally rated as useful (3.9-4.6. on a 5-point Likert scale). Most of the participants reported the programme was a useful introduction into NHS and workforce. |
|  |  |  |  |  |  | Gender: no information |  |  |
|  |  |  |  |  |  | Country of origin: mostly India and Nigeria |  |  |
| Ong & Gayen, 2003 [51] | United Kingdom/ London | Refugee doctors | Mixed Methods | This programme consisted primarily of clinical practise and was complemented by an induction day and an educational supervisor. | N = 29 | Age: mean male 32 / mean female 36 | Self-developed questionnaires at the end of the programme and analysis of discussions | All participants rated the scheme to be good or excellent (26/29). Most of the participants reported an increase in self-esteem and the feeling of belonging to a group. 17 of 29 doctors found a medical employment within 8 months. |
|  |  |  |  |  |  | Gender: 9 female, 20 male |  |  |
|  |  |  |  |  |  | Country of origin: Iraq (n = 14), Afghanistan (n = 5), Algeria (n = 2), Iran (n = 2), Uganda/Congo/Russia/Libya/Ethiopia (each n = 1) |  |  |
| Porter et al., 2008 [52] | United States, Omaha, Nebraska | International medical graduates (IMGs) | Mixed Methods | This programme alternated between theoretical approaches and clinical attachments. Furthermore it gave an orientation into residency and offered social support. | N = 11 (pre-post-test) N = 5 (interviews) | Age: no information | Medical knowledge and skills assessment through self-developed questionnaires pre and post course and interviews after the course | There was a significant increase in post-test scores for medical knowledge and skills such as discharge script writing and Subjective, Objective, Assessment, Plan (SOAP) note definition (p<0.05). Having a respectful and helpful instructor was emphasized by participants as well as their familiarisation with staff and health care. |
|  |  |  |  |  |  | Gender: 3 female, 8 male |  |  |
|  |  |  |  |  |  | Country of origin: mostly India |  |  |
| Romem & Benor, 1993  [27] | Israel | Immigrant doctors | Non-randomised controlled trial | This programme focused on courses on medical subjects through lecturing and problem oriented learning in small groups. Social group activities were integrated. | N = 273 | Age: 25-45 | Success rate in examination compared to a control group | The doctors who participated in the programme had a higher success rate at examination than that of the control group (p<0.019). |
|  |  |  |  |  |  | Gender: 142 female, 131 male |  |  |
|  |  |  |  |  |  | Country of origin: 226 from the Commonwealth Republics (82.8%), 32 Eastern European countries (11.7%) , Rest: South America (5.1%) and one from Iran |  |  |
| Stenerson et al., 2009 [53] | Canada/ Saskatchewan | International medical graduates (IMGs) | Mixed Methods | This programme was based on an induction DVD and an orientation guide. Additionally a two day conference focused on clinical practise issues. | N = 107 | Age: no information | Post-course self-developed questionnaires and telephone interviews post course | Participants were satisfied with conference and 69% reported knowledge gains through conference and media based materials. These materials also supported in adjusting to the new workplace. |
|  |  |  |  |  |  | Gender: no information |  |  |
|  |  |  |  |  |  | Country of origin: no information |  |  |
| Watt et al., 2010 [54] | Canada/ Alberta, Calgary | International medical graduates (IMGs) | Non-randomised controlled trial | This programme combined a didactic course including role plays, case scenarios, practical exercises with a clinical placement including supervision and feedback. | S1: N = 39   S2: N = 235 | Age: S1: range 25-35  S2: M = 39 | Pre-post practicum ITER (S1) and pre-post English language assessment (S1 and S2). Post-course feedback by a self-developed questionnaire. Additionally there was a comparison group on Objective structured clinical examination (OSCE) data and language proficiency (S2) | There were significant changes in the language proficiency (p<0.001) pre and post-test. Improvements were also rated through ITER reports in clinical knowledge and skills (p<0.01). Participants of the programme outperformed other IMGS in their OSCE scores (they passed more OSCE station p<0.05 and had higher scores p.0.01). |
|  |  |  |  |  |  | Gender: S1: 25 female, 14 male; S2: 135 female, 100 male |  |  |
|  |  |  |  |  |  | Country of origin: S1: 17 countries (South American countries, Pakistan, China, Iran and African countries) S2: 22 countries of origin (primarily China, India, Pakistan, Iran, Eastern Europe and African countries) |  |  |
| Higgins et al., 2013 [24] | Australia/ Queensland | Specialist Int. medical graduates | Non-randomised controlled trial | This programme consisted of guided videoconferencing making exam topics a subject of discussion. | N = 166 | Age: no information | Participation and attendance of the media based programme modules associated with exam pass or fail rates | There was an association between tutorial participation and exam success. (Pass rate for those who participated 72%, for those who did not participate 41%). |
|  |  |  |  |  |  | Gender:  no information |  |  |
|  |  |  |  |  |  | Country of origin: no information |  |  |
| Christie et al., 2011 [55] | Australia | International medical graduates (IMGs) | Mixed Methods | This programme consisted of a communication course focusing on language. | N = 8 | Age: no information | Anonymous post course questionnaires, assessment of language skills pre and post programme, focus group post course | There was improvement in pronunciation and non-verbal behaviour. Participants stated the training was useful. |
|  |  |  |  |  |  | Gender: no information |  |  |
|  |  |  |  |  |  | Country of origin: no information |  |  |
