# Supplementary material for: Qualification programmes for immigrant health professionals: A systematic review
Source: PLoS One. 2019 Nov 15;14(11):e0224933. doi: 10.1371/journal.pone.0224933 (PMC6857917; doi:10.1371/journal.pone.0224933)
Supplement: S3 Table — (DOCX) [file pone.0224933.s004.docx]

S3 Table. Quality Assessment

| Qualitative | | | | Quantitative descriptive | | | |
| --- | --- | --- | --- | --- | --- | --- | --- |
| Reference | Kirkpatrick Level | MMAT Items* | Rating MMAT | Reference | Kirkpatrick Level | MMAT Items | Rating MMAT |
| Atack et al., 2012  [20] | 1 and 4 | 1.1. yes  1.2. yes  1.3. yes  1.4. can't tell | 75% | Daniel et al., 2016 [37] | 1 | 4.1. yes 4.2. yes 4.3. yes 4.4. no | 75% |
| McGrath & Henderson, 2009  [40] | 1 | 1.1. yes  1.2. yes  1.3. yes  1.4. yes | 100% | Fernández-Peña, 2012 [38] | 4 | 4.1. yes 4.2. yes 4.3. can't tell 4.4. yes | 75% |
| Gerrish & Griffith, 2004 [46] | 1 | 1.1. yes  1.2. yes 1.3. yes 1.4. no | 75% | Lax et al., 2009 [49] | 1 | 4.1. yes 4.2. no 4.3 yes  4.4 yes | 75% |
| Harris & Delany, 2013 [14] | 1 | 1.1., yes 1.2. yes 1.3. no 1.4. no | 50% |  | | | |
| *MMAT Items:  1. Sources of data relevant to objectives  2. Analysis process relevant to objectives  3. Consideration of findings relate to context  4. Consideration of findings relate to context | | | | *MMAT Items:  1. Sampling strategy relevant to objectives  2. Sample representativeness  3. Measurements appropriate  4. Acceptable response rate | | | |
| Quantitative non randomised | | | | Mixed Methods | | | |
| Reference | Kirkpatrick Level | MMAT Items | Rating MMAT | Reference | Kirkpatrick Level | MMAT Items | Rating MMAT |
| Andrew, 2010 [18] | 3 and 4 | 3.1. no 3.2. yes 3.3. no 3.4. yes | 50% | Lujan & Little 2010 [25] | 4 | 1. 1 yes, 1.2. can't tell, 1.3. no, 1.4. no  4.1. yes, 4.2. yes, 4.3. yes, 4.4. yes 5. 1. yes, 5.2. yes, 5.3. no | 25% |
| Hawken, 2005 [21] | 1, 2, 3 | 3.1. yes 3.2. can't tell 3.3. can't tell  3.4. no | 25% | Ong & Paice, 2006 [15] | 1 and 4 | 1.1 yes, 1.2. yes, 1.3. no, 1.4. no  4.1., yes, 4.2. yes, 4.3. yes, 4.4. yes  5. yes, 5.2. yes, 5.5. no | 50% |
| Majumdar et al. 1999 [39] | 2 | 3.1. no  3.2. yes  3.3. yes 3.4. yes | 75% | Peters & Braeseke, 2016 [19] | 1 and 4 | 1.1.yes, 1.2. yes, 1.3. no, 1.4. no,  4.1.yes, 4.2. yes, 4.3. can't tell, 4.4. yes 5.1. yes, 5.2. yes, 5.3. no | 50% |
| Parrone et al., 2008  [26] | 4 | 3.1. can't tell  3.2. yes 3.3. no 3.4. yes | 50% | Wright et al., 2011 [42] | 1, 2, 3 | 1.1. yes, 1.2. yes, 1.3. yes, 1.4. yes 3.1. no, 3.2. yes, 3.3. no, 3.4. yes 5. 1 yes, 5.2. yes, 5.3. no | 50% |
| Sullivan et al., 2002,  [41] | 2 | 3.1. no 3.2. yes  3.3. no 3.4. yes | 50% | Baker & Robson, 2012 [43] | 1 and 2 | 1. 1. yes, 1.2. yes, 1.3. yes, 1.4. yes 3.1. no, 3.2. no 3.3. no, 3.4. yes 5. yes, 5.2. yes, 5.3. yes | 25% |
| Bruce et al., 1974 [44] | 2 | 3.1. no 3.2. yes 3.3. no  3.4. yes | 50% | Cheung 2011 [45] | 1 | 1.1. yes, 1.2. can’t tell, 1.3. no, 1.4. no 4.1. yes, 4.2. can't tell, 4.3. can't tell, 4.4. yes  5.1. yes, 5.2. yes, 5-3. no | 25% |
| Elis et al., 2005  [22] | 1 and 4 | 3.1. can't tell 3.2. yes  3.3. yes 3.4. yes | 75% | Greig et al., 2013 [23] | 1, 2, 4 | 1.1.yes, 1.2. can't tell, 1.3. can't tell, 1.4. no  3.1. can't tell, 3.2. yes, 3.3. yes, 3.4. yes  5. yes, 5.2. yes, 5.no | 25% |
| Goldszmidt et al., 2007 [47] | 1 and 2 | 3.1. yes  3.2. no 3.3. no 3.4. yes | 50% | Ong & Gayen, 2003 [51] | 1 and 4 | 1.1 yes, 1.2. can't tell, 1.3. no, 1.4. no 3. 1 can't tell, 3.2. no, 3.3. can't tell, 3.4. yes  5.1. yes, 5.2. yes, 5.3. no | 25% |
| Horner, 2004 [48] | 1 | 3.1. yes 3.2. no 3.3. can't tell 3.4. no | 25% | Porter et al., 2008 [52] | 1, 2, 3 | 1.1. yes, 1.2. yes, 1.3. no, 1.4. can't tell 4.1. yes, 4.2. yes, 4.3. yes, 4.4. yes 5.1. yes, 5.2. yes, 5.2. no | 50% |
| Ong et al., 2002 [50] | 1 | 3.1. no  3.2. yes  3.3. can't tell 3.4. yes | 50% | Stenerson et al., 2009 [53] | 1 | 1.1. yes, 1.2. can't tell, 1.3. no, 1.4. no  3. 1 can't tell, 3.2. yes, 3.3. can't tell, 3.4. yes  5.1 yes, 5.2 yes, 5.3 no | 25% |
| Romem & Benor, 1993 [27] | 4 | 3.1 no  3.2. yes 3.3. no  3.4. yes | 50% | Christie et al., 2011 [55] | 1 and 2 | 1.1. yes, 1.2. no, 1.3. no, 1.4. no 3. 1 can't tell, 3.2. yes, 3.3. can't tell, 3.4. yes  5.1. yes, 5.2. yes, 5.3. no | 25% |
| Watt et al., 2010 [54] | 1 and 2 | S1: 3.1. can't tell  3.2. yes 3.3. can't tell  3.4. yes  S2: 3.1. can't tell 3.2. yes 3.3 can't tell 3.4. yes | 50%  50% |  | | | |
| Higgins et al., 2013  [24] | 4 | 3.1. can't tell 3.2. yes 3.3. can't tell 3.4. yes | 50% |  |  |  |  |
| *MMAT Items:  1. Low-biased way of recruiting  2. Measurements appropriate  3. Consideration of differences between groups  4. Complete outcome data | | | | *MMAT Items:  1. Mixed methods research design relevant to objectives  2. Integration of results relevant to objectives  3. Consideration of limitations associated with this integration | | | |
